# Does Molecular Profiling of *KRAS*-Mutant Non-Squamous Non-Small Cell Lung Cancer (NSCLC) Help in Treatment Strategy Planning?

**DOI:** 10.3390/curroncol29070379

**Published:** 2022-07-07

**Authors:** Nagla Abdel Karim, Asad Ullah, Peterson Pathrose, Hassana Fathallah, Ashley Perry, John C. Morris, Jiang Wang, Sandra L. Starnes

**Affiliations:** 1 Inova Schar Cancer Institute, Department of Medicine, University of Virginia, Fairfax, VA 22031, USA; 2 Department of Pathology, Vanderbilt University Medical Center, Nashville, TN 37232, USA; drasadkhankakar@gmail.com; 3 Department of Surgery, University of Cincinnati, Cincinnati, OH 45221, USA; peterpathrose@yahoo.com (P.P.); sandra.starnes@uc.edu (S.L.S.); 4 Department of Medicine, Hematology and Oncology University of Cincinnati, Cincinnati, OH 45221, USA; hassana.fathallah@uc.edu (H.F.); ashley.perry@uc.edu (A.P.); jcmorris16@outlook.com (J.C.M.); 5 Department of Pathology and Laboratory Medicine, University of Cincinnati, Cincinnati, OH 45221, USA; wajn@ucmail.uc.edu

**Keywords:** systemic therapy, mutations, molecular profiling

## Abstract

Background: Several studies suggest that patients with *KRAS*-mutant NSCLC fail to benefit from standard systemic therapies and do not respond to EGFR inhibitors. Most recently, *KRAS* 12c data suggest specific treatment for improving ORR and OS. There is a clear need for therapies specifically developed for these patients. Moreover, data that might be suggestive of a response to specific therapies, such as BRCA1, are needed, and two mutations that were studied in other malignancies show more response to PARP inhibitors. Molecular profiling has the potential to identify other potential targets that may provide better treatment and novel targeted therapy for KRAS-mutated NSCLC. Methods: We purified RNA from archived tissues of patients with stage I and II NSCLC with wild-type (*wt*) and mutant (*mt*) *KRAS* tumors; paired normal tissue adjacent to the tumor from 20 and 17 patients, respectively, and assessed, using real-time reverse transcriptase–polymerase chain reaction (RT-PCR), the expression of four genes involved in DNA synthesis and repair, including thymidylate synthase (TS), BRCA1, ECCR1, RAP80, and the proto-oncogene SRC. Additionally, we assessed the expression of PD-L1 in *mt* *KRAS* tumors with immunohistochemistry using an antibody against PD-L1. Results: Our results show that in *mt* *KRAS* tumors, the level of expression of ERCC1, TS, and SRC was significantly increased in comparison to paired normal lung tissue (*p* ≤ 0.04). The expression of BRCA1 and RAP80 was similar in both *mt* *KRAS* tumors and paired normal tissue. Furthermore, the expression of BRCA1, TS, and SRC was significantly increased in *wt* *KRAS* tumors relative to their expression in the normal lung tissue (*p* < 0.044). The expression of ERCC1 and RAP80 was similar in *wt* *KRAS* tumors and paired normal tissue. Interestingly, SRC expression in *mt* *KRAS* tumors was decreased in comparison to *wt* *KRAS* tumors. Notably, there was an expression of PD-L1 in the tumor and stromal cells in a few (5 out of 20) *mt* *KRAS* tumors. Our results suggest that a greater ERCC1 expression in *mt* KRAS tumors might increase platinum resistance in this group of patients, whereas the greater expression of BRCA1 in *wt* *KRAS* tumor might be suggestive of the sensitivity of taxanes. Our data also suggest that the combination of an SRC inhibitor with a TS inhibitor, such as pemetrexed, might improve the outcome of patients with NSCLC and in particular, patients with *wt* *KRAS* tumors. PD-L1 expression in tumors, and especially stromal cells, suggests a better outcome. Conclusion: *mt* *KRAS* NSCLC patients might benefit from a treatment strategy that targets KRAS in combination with therapeutic agents based on pharmacogenomic markers, such as SRC and BRCA1. *mt* *KRAS* tumors are likely to be platinum-, taxane-, and pemetrexed-resistant, as well as having a low level of PD-L1 expression; thus, they are less likely to receive single-agent immunotherapy, such as pembrolizumab, as the first-line therapy. *wt* *KRAS* tumors with BRCA1 positivity tend to be sensitive to taxane therapy and, potentially, platinum. Our results suggest the need to develop targeted therapies for *KRAS*-mutant NSCLC or combine the targeting of oncogenic *KRAS* in addition to other therapeutic agents specific to the molecular profile of the tumor.

## 1. Introduction

The cloning and studies of the normal human genes homologs of the rat Harvey-(*HRAS*) reveal that the mutation in oncogenic alleles is a result of a single-point mutation in *KRAS* and neuroblastoma-RAS (*NRAS*). This discovery laid the foundations for the study by Santos et al. who first reported *mt KRAS* in lung cancer, which was later confirmed [1,2,3]. While the link between lung cancer and *KRAS* mutation (*mt KRAS*) is a widely discussed topic in research, attempts to develop targeted therapy are reported to be elusive [4,5]. The failure to yield positive results may be, in part, because the *KRAS* gene belongs to the large RAS gene family, which was originally discovered during the study of retroviruses causing cancer in animals in the early 1960s [6] and encodes a family of membrane-bound, 21-Kd, GTP-binding proteins involved in the regulation of key processes, such as cell growth, differentiation, and apoptosis. This activity is achieved through interacting with multiple effectors, including those in the MAPK, STAT, and PI3K signaling cascades [7,8]. After the single-point mutation, *KRAS* protein produces defective activity from the GTPase; targeting *KRAS* by itself is challenging; therefore, targeting its effector pathways may be a better option.

Subsequently, when targeted therapy was investigated in a specific subset of *KRAS* mutations, promising results were achieved. Recently, Govindan et al. reported an ORR of 54% (all partial responses), with 46% of patients achieving stable disease, and a DCR of 100% when targeting *KRAS* G12C (AMG 510) [9].

The Kirsten rat sarcoma viral oncogene homolog (*KRAS*) is the most common mutated oncogene in non-small cell lung carcinoma. In Western countries, the *KRAS* mutations are found in approximately 25% of NSCLCs [10] and are most frequently observed in exon 2 (codons 12 and 13) and, less commonly, in exon 13 (codon 61) [11]. *KRAS* mutations are typically clonal, occur in early carcinogenesis, and are mutually exclusive with other mutations in lung cancer such as *EGFR* and *ALK* [12]. The most frequent *KRAS* amino acid mutations in NSCLC are guanine to thymine or guanine to cytosine. The glycine 12 to cysteine (G12C) accounts for 41% of patients, followed by glycine 12 to valine (G12V); both mutations are associated with a tobacco-smoking history. Whereas mutations involving guanine to adenine nucleotide changes, such as glycine 12 to aspartic acid (G12D), are more common in non-smokers [11]. It has also been noted that about 50% of *mt KRAS* has co-mutations in NSCLC. The most frequent non-oncogenic co-mutations are *TP53* (40%), serine/threonine kinase 11 (STK11) (20%), and Kelch-like ECH-associated protein 1 (KEAP1) (13%) [13]. The understanding of the ultrastructure of oncogenic mutations in KRAS pathways led to the devolvement of allele-specific inhibitors for *mt KRAS* in NSCLC [10]. 

*BRCA1* and *BRCA2* are tumor suppressor genes involved in cell cycle regulation, including replication, mitotic spindle assembly, and apoptosis. Their regulatory role also affects transcription and DNA damage response (DDR) [14,15]. *BRCA*-mutated individuals have an impaired ability to repair DNA. Thus carriers of mutated *BRCA* are more sensitive to therapies that induce DNA damage, such as platinum-based chemotherapy and radiotherapy [16]. Poly (ADP-ribose) polymerase (PARP) inhibitors (PARPi) are the first approved agents to target DNA repair in cancer. PARP inhibitors changed therapeutic strategies by targeting DNA repair in various cancers, including breast, pancreatic, ovarian, and prostate cancers [17,18,19]. *BRCA1* and *BRCA2* mutations occur in 5–10% of NSCLC. These mutations lead to the inactivation of homologous recombination (HR)-mediated DNA repair [20]. Normal lung tissue does not harbor germline *BRCA1/2* mutations; however, due to the high mutational burden found in smokers, 5–10% of NSCLC patients exhibit somatic *BRCA1* and *BRCA2* mutations [20].

Excision repair cross-complementing group 1 (*ERCC1*) is a component of the nucleotide excision repair (NER) pathway. *ERCC1* functions in the repair of DNA strand breaks caused by platinum-based drugs, thus conferring an increased resistance to platinum-based therapy in lung cancer [21]. *ERCC1* together with *ERCC4* (XPF) act in a rate-limiting multistep process in NER and DNA repair. The down-regulation of *ERCC1* is associated with increased chemotherapy sensitivity and increased responses to platinum-based chemotherapeutic agents in lung cancer [22]. *ERCC1* single-nucleotide polymorphism (SNPs), including s11615 and rs3212986, have been studied to assess cancer risk. Chen et al. demonstrated that smokers with the *ERCC1* rs11615 TT genotype had a 1.8-fold increased risk of developing lung cancer [22].

Thymidylate synthase (TS) is the rate-limiting enzyme in DNA synthesis. TS expression is more pronounced in metabolically active sites such as the nucleus and mitochondria. The inhibition of TS activity within cancer cells makes TS a target for anticancer therapies [23]. Studies have shown conflicting data on TS expression and prognosis. A study by Kulda et al. revealed that TS is highly expressed in NSCLC tumor tissue in comparison to normal lung tissue. The results of their study show that patients who received platinum-based adjuvant chemotherapy in combination with paclitaxel or gemcitabine had shorter disease-free intervals and overall survival rates than patients with a high expression of TS in NSCLC [24].

## 2. Materials and Methods

### 2.1. Study Population

**Figure 1:**
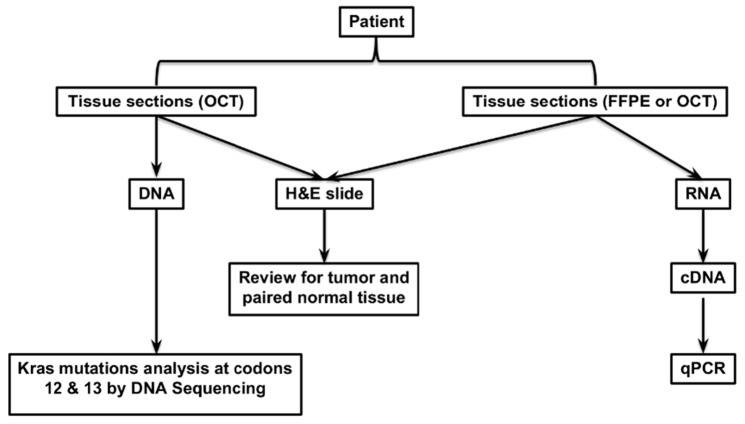
The study protocol for patient selection, tissue extraction for molecular analysis, and PD-L1 expression.

Patients with known or suspected lung cancer that were enrolled in the Thoracic Tumor Registry at the University of Cincinnati were studied. The Institutional Review Board (IRB) approved the protocol, and informed consent was obtained from all patients. Tumors, normal lung tissue, clinical and outcome data were prospectively collected. All patients with stage I and II non-small cell lung cancer (adenocarcinomas) who underwent curative resection by a dedicated thoracic surgical oncologist between 1 March 2006 and 30 April 2012 were included. Patients who received neoadjuvant chemotherapy or radiation were excluded. A portion of resected tumor specimens along with normal lung tissue was placed in RNA stabilization solution (ThermoFisher Scientific, Waltham, MA, USA), snap-frozen and stored in an OCT compound as a frozen section or fixed in 10% formalin and paraffin-embedded. The study protocol and tissue extraction for PD-L1 expression with DNA extraction and KRAS mutational analysis are detailed in Figure 1. 

### 2.2. DNA Extraction from Paraffin Tissue Blocks

Serial 10 µm sections were cut from formalin-fixed, paraffin-embedded lung tissue (tumor or normal lung tissue) onto a glass slide. A simultaneous section was hematoxylin-eosin (H&E)-stained to determine tumor and normal tissue. The tumor was micro-dissected into an Eppendorf tube, deparaffinized in xylene, and further washed with 100% ethanol. The supernatant was removed after centrifugation, and the residual tissue (tumor or normal) was incubated in tissue lysis buffer with 20 µg of Proteinase K (Qiagen, Germantown, MD, USA). DNA was extracted using a Qiagen Purification kit as per the manufacturer’s protocol. The concentration of the DNA was determined using a Nanodrop spectrophotometer ND-1000.

### 2.3. KRAS Mutation at Codons 12 and 13 

PCR was performed on 90 µL containing 6 µL DNA, 20 µM forward and reverse primer mixed with True Allele Premix (Applied Biosystems) at an annealing temperature of 55 °C. The primers set for codon 12 and 13 of the KRAS gene were (forward) 5′ GGT ACT GGT GGA GTA TTT GAT AGT G 3′ and (Reverse) 5′ AAA GAA TGG TCC TGC ACC 3′. The PCR product was separated on 2% agarose gel to confirm the product size. The PCR product was further purified using ExoSAP-IT (USB Corporation, Cleveland, OH, USA) to detect KRAS mutation using an automatic DNA sequencer (Applied Biosystems, Foster City, CA, USA). 

### 2.4. Immunohistochemistry

The formalin-fixed, paraffin-embedded tissues were sectioned to 4 μm and deparaffinized. Antigen retrieval was carried out using a 10 mM citrate buffer maintained at pH 6. The sections were then incubated in 0.5% of hydrogen peroxide in methanol for 15 min to block endogenous peroxidase activity. The sections were incubated with a PD-L1 rabbit monoclonal antibody (Cell Signaling, Danvers, MA, USA) at 4 °C overnight. The slides were washed in 1X PBS and incubated at room temperature for 1 h with 1:200 dilution of biotinylated anti-rabbit secondary antibody (Vector, Burlingame, CA, USA). The slides were washed in 1X PBS and incubated in ABC (Vector Laboratories, Burlingame, CA, USA) for 30 min. The sections were then developed for color using 3-3′-diaminobenzidine (Vector Laboratories, Burlingame, CA, USA). 

### 2.5. To Evaluate the Level of PD-L1 Expression in the Stroma, the Percentage of Positively

Stained stromal cells were identified, and the staining intensity was graded on a scale of 0 (no staining), 1 (weak staining), 2 (moderate staining), and 3 (intense staining).

For each sample, the staining percentage and staining intensity scores were multiplied together to give the stromal staining index.

### 2.6. RNA Extraction from Tissue Blocks

An H&E stain from each specimen was obtained and reviewed by a pathologist to accurately select blocks with a high percentage of tumor cells and normal tissue. Only tumor samples containing more than 60% of tumor tissue using the H&E-stained section were analyzed. Three to five adjacent unstained slides of 10 μm were obtained from the corresponding block for the extraction of total RNA. Total RNA was extracted from FFPE tissue sections with a Qiagen RNeasy FFPE Kit and from OCT tissues sections with an Agilent Absolutely RNA FFPE Kit, following the manufacturers’ protocols. The concentration of RNA was assessed.

### 2.7. Quantitative RT-PCR Analysis

**Figure 2:**
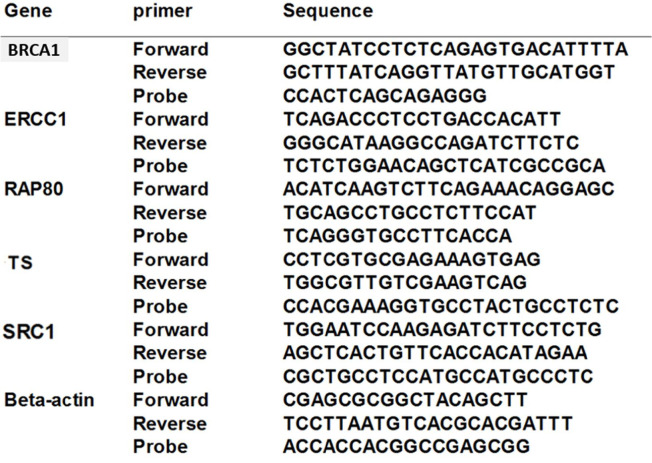
Primers and probes used in quantitative reverse transcriptase–polymerase chain reaction (qRT-PCR).

Complementary DNA (cDNA) was synthesized using oligo dT and random hexamer primers. Aliquots of cDNA were used for quantitative real-time PCR in triplicates to assess the expression of thymidylate synthase (TS), BRCA1, ECCR1, RAP80, and the proto-oncogene SRC (Figure 2). The relative expression levels of the studied genes were normalized to the expression of the housekeeping gene b-actin. The data are expressed as a relative unit and as fold change compared to normal tissue.

### 2.8. Statistical Analysis 

The Student’s *t*-test was used to determine statistical significance. *p* ≤ 0.05 was considered statistically significant. 

## 3. Results

**Figure 3:**
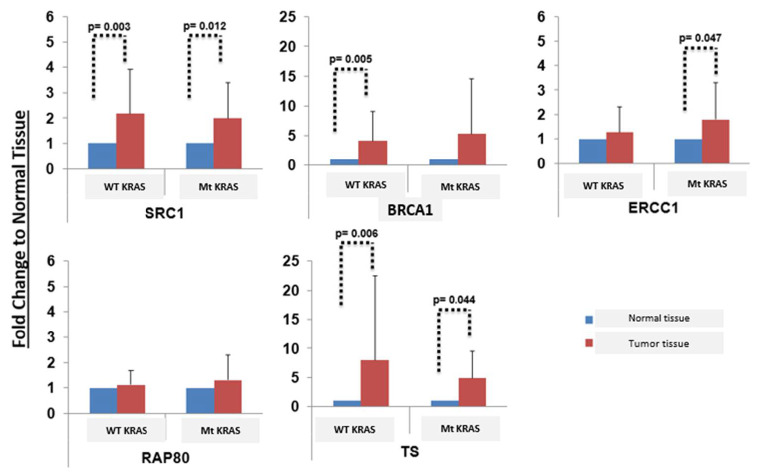
The expression of SRC, BRCA1, RAP80, ERCC1, and TS in normal tissue and tumor tissue with mt *KRAS* and wt *KRAS* (fold change to normal tissue).

**Figure 4:**
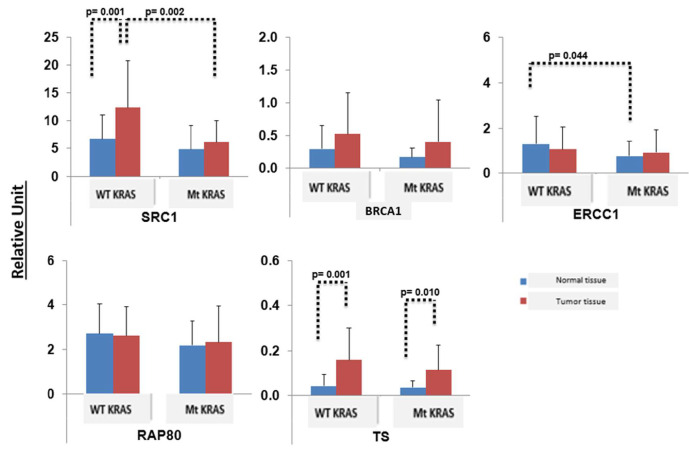
The expression of SRC, BRCA1, RAP80, ERCC1, and TS in normal tissue and tumor tissue with mt *KRAS* and wt *KRAS* (relative unit).

In *mt KRAS*-positive tumors, the levels of expression of ERCC1, TS, and SRC were significantly increased in comparison to paired normal lung tissue (*p* < 0.04). Meanwhile, BRCA1 and RAP80 expressions showed no significant differences in both *mt* *KRAS* tumors and the paired normal tissue. Furthermore, the expression of the BRCA1, TS, and SRC genes was significantly increased in *wt KRAS* tumors relative to their expression in the normal lung tissue (*p* < 0.044). Expressions of *ERCC1* and *RAP80* were similar in *wt KRAS* tumors and with paired normal tissue. Interestingly, SRC expression was reduced in *mt KRAS* tumors compared to *wt* *KRAS* tumors (Table 1, Figure 3 and Figure 4).

PD-L1 Expression Analysis

Positive PD-L1 protein expression was observed in five patients (25%) in tumor-infiltrating lymphocytes/immune cells. Tumor cells display a cytoplasmic staining pattern.

The programmed death ligand inhibitor protein expression is classified into four categories.

**Figure 5:**
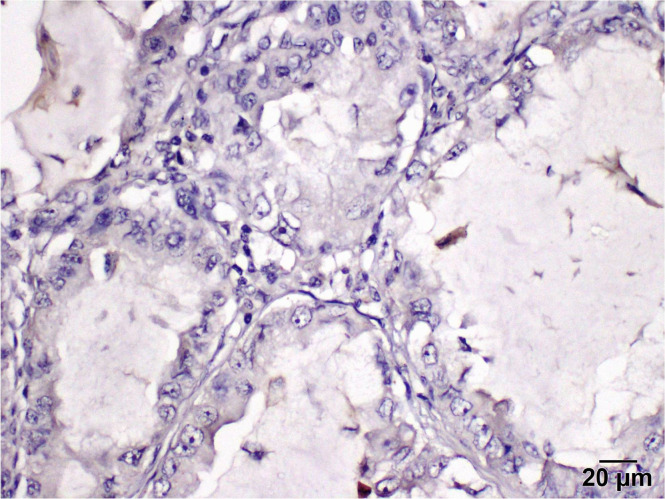
Immunohistochemistry demonstrates a negative staining pattern for PD-L1 expression (400×).

**Figure 6:**
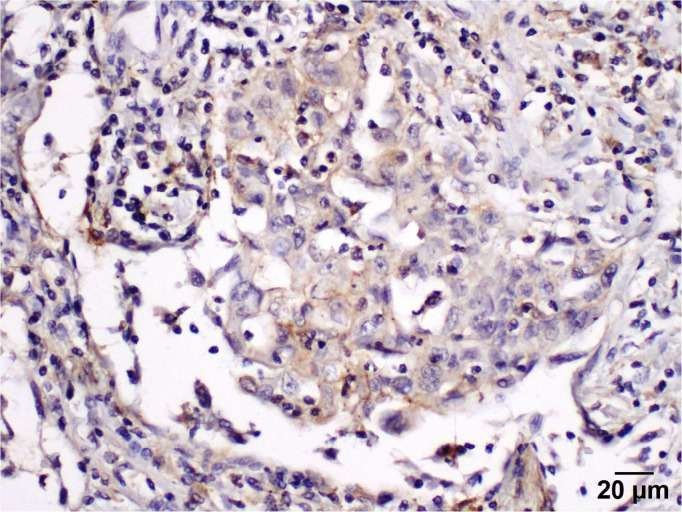
Immunohistochemistry demonstrates a focal (weak) staining pattern for PD-L1 expression (400×).

**Figure 7:**
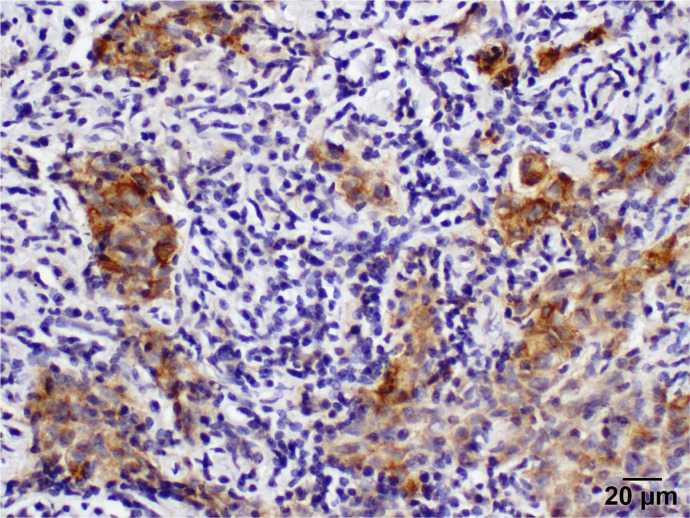
Immunohistochemistry demonstrates a strong staining pattern of PD-L1 expression (400×).

1. Negative (−)with staining of less than 1% of tumor cells (Figure 5).
2. Weak positive (+) with staining of 2–10% of tumor cells
3. Moderate positive (++) with staining of 11–50% of tumor cells (Figure 6).
4. Strong positive (+++) with diffused staining in more than 60% of tumor cells (Figure 7).

## 4. Discussion

Despite major advances in cancer treatment, platinum-based therapy has remained the cornerstone in the treatment of advanced NSCLC [25,26,27,28,29]. However, *mt KRAS* lung cancer was found to be particularly resistant to such therapies, hence the need to develop a wider molecular profile and tailored therapies for this group of patients. Targeted approaches to treatment can be more successful in terms of survival advantage and improvements in quality of life as it can replace cytotoxic conventional chemotherapy [30,31]. In a retrospective study conducted on 47 NSCLC patients, researchers found that *mt* *KRAS* NSCLC had a significantly lower expression of BRCA1 and TS proteins. When comparing their results in wild-type NSCLC patients, however, SRC expression showed no significant changes between *mt* and *wt* *KRAS* tumors [30]. Although BRCA 1 overexpression is associated with treatment resistance in NSCLC [22], our results reveal similar expressions of BRCA1 in both *mt* KRAS tumors and paired normal tissue. Both studies’ results conclude that the poor response of mt *KRAS* NSCLC to treatment is independent of BRAC1 expression. A low expression of receptor-associated protein 80 (RAP80) was found to have a positive impact on the survival of NSCLC patients, and both BRCA1 and RAP80 had low expression levels in patients, showing an increased benefit from platinum therapy [32,33].

Conversely, levels of ERCC1 expression were significantly increased in comparison to paired normal lung tissue (*p* < 0.04). ERCC1 expression was repeatedly linked to resistance to platinum therapy [34,35], which suggests that it can be linked to the poor response of mt *KRAS* tumors to platinum therapy [3].

Although KRAS activation was linked to SRC overexpression in pancreatic carcinoma, the same study failed to establish a link between *mt KRAS* and SRC overexpression [36]. Our patients had a significantly increased SRC expression in mt *KRAS* compared to paired normal tissue (*p* < 0.04). While the number of patients is limited, this finding should be further investigated as SRC is a druggable target that can offer a treatment option in mt *KRAS* patients. TS and SRC overexpression were previously linked to each other, and it was reported that pemetrexed resistance emerges as a result of TS overexpression. Although the link between the TS and SRC pathways is not yet clear, TS overexpression is associated with concomitant SRC overexpression, leading to the hypothesis that using TKIs can block SRC and consequently, lead to TS overexpression. The results have been validated in cell lines but not in human subjects thus far [31].

Remarkably, prolonged survival was noted in patients whose samples expressed increased PD-L1 in tumor cells in contrast to patients with decreased or no expression [37]. Our results are consistent with other studies in suggesting that patients with robust PD-L1 protein expression are expected to respond better to immunotherapy [38].

In the phase 2 trial, patients with *KRAS*-G12C-mutated NSCLC were previously treated with standard therapies such as platinum-based chemotherapy and program death inhibitors (PD-1) or program death-ligand inhibitors (PD-L1). These patients received selective reversible inhibitors of the G12C-activated *KRAS* oncogene, such as sotorasib. In this multicenter study, the objective response was evaluated by the radiological review of the patients. The results show that 3.2% of patients showed a complete response to sotorasib, and 34% of patients had a partial response to the *KRAS* G12C inhibitor. The median progression-free survival was 7 months, the median duration of response was 11.1 months, and the overall survival was 12.5 months [39]. The current standard therapies for advanced NSCLC include immune checkpoint inhibitors with combination chemotherapy or immunotherapy alone in patients with newly diagnosed *KRAS*-mutated NSCLC [40]. Disease progression in patients after immunotherapy treatment is generally treated with single-agent chemotherapy such as docetaxel or pemetrexed; however, the response rate in these patients is less than 10% [41]. In the REVEL and LUME-Lung 1 trials, the survival rate was longer with the addition of ramucirumab, a monoclonal antibody targeting the vascular endothelial growth factor receptor, or nintedanib, a broadly acting tyrosine kinase inhibitor [42]. In this phase 2 trial, patients with advanced NSCLC previously treated with both platinum-based chemotherapy and immune checkpoint inhibitors showed a rapid and durable response to sotorasib. The response to sotorasib was observed in all PD-L1 expression levels [39]. *KRAS* inhibitors, such as adagrasib and sotorasib, showed promising results in patients with advanced NSCLC. The mechanism of resistance to these therapies is largely unknown. A study by Awad et al. revealed a putative mechanism for resistance to adagrasib. In this study, acquired *KRAS* alterations included G12D/R/V/W, G13D, Q61H, R61H, Y96C, and H95D/Q/R. Amplification in the expression of the *KRAS* G12C allele was also observed. Other bypass mechanisms for resistance included MET amplification, activating mutations in *NRAS, BRAF, MAP2K1*, and *RET*. Several oncogenic fusions were observed involving *ALK, RET, BRAF*, *RAF1*, and *FGFR*3. Loss-of-function mutations were also observed in *NF1* and *PTEN.* Thus, this study reveals diverse genomic mechanisms which impart resistance to *KRAS* G12C [43].

## 5. Limitations

The tissue for this study was collected from the biorepository tissue bank of the University of Cincinnati, OH, USA and approved by the IRB committee. However, the clinical data of the patients were provided for this study, which is its main limitation. The tissue used for this study was exhausted; unfortunately, no further molecular testing can be carried out.

## 6. Conclusions

Our results suggest that *mt KRAS* NSCLC patients might benefit from a treatment strategy that targets KRAS in combination with therapeutic agents based on pharmacogenomic markers such as SRC and BRCA1 expression levels. SRC expression has been described as a potential resistance mechanism to pemetrexed-based therapy. *BRCA1* expression is related to responsiveness to taxane-based therapy. Both pemetrexed- and taxane-based therapies are utilized in the treatment of patients with *KRAS* mutations. Only, *KRAS* G12c has an approved targeted therapy. Future studies that focus on the selection of chemotherapeutic agents in combination with *KRAS*-targeted therapies are needed.

## Figures and Tables

**Table 1:** The table illustrates the mutations found in *wt KRAS*, *mt KRAS,* and normal lung tissue.

Oncogene.	ERCC1	BRCA1	TS	SRC	PD-L1
*mt KRAS*	+	−	+	−	25%
*wt* *KRAS*	−	+	+	+	
Normal lung tissue	−	−	−	−	

## Data Availability

The data presented in this study are available on request from the corresponding author.

## References

[1] Santos E., Martin-Zanca D., Reddy E.P., Pierotti M.A., Porta G., Barbacid M. (1983). Malignant activation of a K-ras oncogenein ling carcinoma but not in normal tissue of the same patient. Science.

[2] Ferrer I., Zugazagoitia J., Herbertz S., John W., Paz-Ares L., Schmid-Bindert G. (2018). KRAS-Mutant non-small cell lung cancer: From biology to therapy. Lung Cancer.

[3] Karim N.A., Starnes S., Morris J., Pathrose P., Perry A., Fathallah H. (2014). Abstract A01: KRAS molecular profiling in non-squamous non-small cell lung cancer (NSCLC). Clin. Cancer Res..

[4] Bhattacharya S., Socinski M.A., Burns T.F. (2016). *KRAS* mutant lung cancer: Progress thus far on an elusive therapeutic target. Clin. Transl. Med..

[5] Zhang J., Park D., Shin D.M., Deng X. (2015). Targeting KRAS-mutant non-small cell lung cancer: Challenges and opportunities. Acta Biochim. Biophys. Sin..

[6] Riely G.J., Marks J., Pao W. (2009). KRAS Mutations in Non-Small Cell Lung Cancer. Proc. Am. Thorac. Soc..

[7] Downward J. (1998). New exchange, new target. Nature.

[8] Shields J.M., Pruitt K., McFall A., Shaub A., Der C.J. (2000). Understanding Ras: ‘It ain’t over ’til it’s over’. Trends Cell Biol..

[9] Govindan R., Fakih M., Price T., Falchook G., Desai J., Kuo J., Strickler J., Krauss J., Li B., Denlinger C. Phase I study of safety, tolerability, PK and efficacy of AMG 510, a novel KRAS G12C inhibitor, evaluated in NSCLC. Proceedings of the IASLC 2019 World Conference on Lung Cancer hosted by the International Association for the Study of Lung Cancer.

[10] de la Fuente E.C., Garcia M.E.O., Rueda A.G., Lage Y., Garrido P. (2022). Targeting KRAS in Non-Small Cell Lung Cancer. Front. Oncol..

[11] Friedlaender A., Drilon A., Weiss G.J., Banna G.L., Addeo A. (2020). KRAS as a druggable target in NSCLC: Rising like a phoenix after decades of development failures. Cancer Treat. Rev..

[12] Reck M., Carbone D., Garassino M., Barlesi F. (2021). Targeting KRAS in non-small-cell lung cancer: Recent progress and new approaches. Ann. Oncol..

[13] Scheffler M., Ihle M.A., Hein R., Merkelbach-Bruse S., Scheel A.H., Siemanowski J., Brägelmann J., Kron A., Abedpour N., Ueckeroth F. (2019). K-ras Mutation Subtypes in NSCLC and Associated Co-occuring Mutations in Other Oncogenic Pathways. J. Thorac. Oncol..

[14] Oh M., McBride A., Yun S., Bhattacharjee S., Slack M., Martin J.R., Jeter J., Abraham I. (2018). *BRCA1* and *BRCA2* Gene Mutations and Colorectal Cancer Risk: Systematic Review and Meta-analysis. JNCI J. Natl. Cancer Inst..

[15] Lee Y.C., Lee Y.C., Li C.Y., Lee Y.L., Chen B.L. (2020). BRCA1 and BRCA2 gene mutations and lung cancer risk: A meta-analysis. Medicina.

[16] Li S., Tao L., Dai H., Gong X., Zhuo Y., Xiang H., Zhao Y., Gao Q., Deng L. (2021). BRCA1 Versus BRCA2 and PARP Inhibitors Efficacy in Solid Tumors: A Meta-Analysis of Randomized Controlled Trials. Front. Oncol..

[17] Coleman R.L., Fleming G.F., Brady M.F., Swisher E.M., Steffensen K.D., Friedlander M., Okamoto A., Moore K.N., Efrat Ben-Baruch N., Werner T.L. (2019). Veliparib with First-Line Chemotherapy and as Maintenance Therapy in Ovarian Cancer. N. Engl. J. Med..

[18] Han H.S., Diéras V., Robson M., Palácová M., Marcom P.K., Jager A., Puhalla S. (2018). Veliparib with temozolomide or carboplatin/paclitaxel versus placebo with carboplatin/paclitaxel in patients with BRCA1/2 locally recurrent/metastatic breast cancer: Randomized phase II study. Ann. Oncol..

[19] Golan T., Hammel P., Reni M., Van Cutsem E., Macarulla T., Hall M.J., Park J.-O., Hochhauser D., Arnold D., Oh D.-Y. (2019). Maintenance Olaparib for Germline BRCA-Mutated Metastatic Pancreatic Cancer. N. Engl. J. Med..

[20] Diossy M., Sztupinszki Z., Borcsok J., Krzystanek M., Tisza V., Spisak S., Rusz O., Timar J., Csabai I., Fillinger J. (2021). A subset of lung cancer cases shows robust signs of homologous recombination deficiency associated genomic mutational signatures. NPJ Precis. Oncol..

[21] Rao D., Mallick A.B., Augustine T., Daroqui C., Jiffry J., Merla A., Chaudhary I., Seetharam R., Sood A., Gajavelli S. (2019). Excision repair cross-complementing group-1 (ERCC1) induction kinetics and polymorphism are markers of inferior outcome in patients with colorectal cancer treated with oxaliplatin. Oncotarget.

[22] Chen L.-H., Shen T.-C., Li C.-H., Chiu K.-L., Hsiau Y.-C., Wang Y.-C., Gong C.-L., Wang Z.-H., Chang W.-S., Tsai C.-W. (2020). The Significant Interaction of Excision Repair Cross-complementing Group 1 Genotypes and Smoking to Lung Cancer Risk. Cancer Genom. Proteom..

[23] Li X.-Y., Wang D.-P., Lu G.-Q., Liu K.-L., Zhang T.-J., Li S., O Mohamed K., Xue W.-H., Qian X.-H., Meng F.-H. (2020). Development of a novel thymidylate synthase (TS) inhibitor capable of up-regulating P53 expression and inhibiting angiogenesis in NSCLC. J. Adv. Res..

[24] Kulda V., Hrda K., Houdek Z., Dobra J.K., Vrzakova R., Svaton M., Safranek J., Dolezal J., Babuska V., Pesek M. (2017). Predictive Significance of Thymidylate Synthase Expression in Non-small Cell Lung Cancer. Anticancer Res..

[25] Karim N.A., Schuster J., Eldessouki I., Gaber O., Namad T., Wang J., Xie C., Morris J.C. (2017). Pulmonary sarcomatoid carcinoma: University of Cincinnati experience. Oncotarget.

[26] Hassan R., Gulati S., Mahender Y., Eldessouki I., Siddiqi N.I., Xie C., Pruemer J., Karim N.A. (2017). Impact of Low Molecular Weight Heparin on Overall Survival in Patients with Advanced Lung Cancer: A Retrospective Study. Am. J. Clin. Exp. Med..

[27] Hotta K., Matsuo K., Ueoka H., Kiura K., Tabata M., Tanimoto M. (2004). Addition of platinum compounds to a new agent in patients with advanced non-small-cell lung cancer: A literature based meta-analysis of randomised trials. Ann. Oncol..

[28] Fossella F., Pereira J.R., Von Pawel J., Pluzanska A., Gorbounova V., Kaukel E., Mattson K.V., Ramlau R., Szczęsna A., Fidias P. (2003). Randomized, Multinational, Phase III Study of Docetaxel Plus Platinum Combinations Versus Vinorelbine Plus Cisplatin for Advanced Non–Small-Cell Lung Cancer: The TAX 326 Study Group. J. Clin. Oncol..

[29] D’Addario G., Pintilie M., Leighl N.B., Feld R., Cerny T., Shepherd F.A. (2005). Platinum-Based Versus Non-Platinum-Based Chemotherapy in Advanced Non-Small-Cell Lung Cancer: A Meta-Analysis of the Published Literature. J. Clin. Oncol..

[30] Monica V., Iacono M.L., Bracco E., Busso S., di Blasio L., Primo L., Peracino B., Papotti M., Scagliotti G. (2016). Dasatinib modulates sensitivity to pemetrexed in malignant pleural mesothelioma cell lines. Oncotarget.

[31] Qian X.-L., Zhang J., Li P.-Z., Lang R.-G., Li W.-D., Sun H., Liu F.-F., Guo X.-J., Gu F., Fu L. (2017). Dasatinib inhibits c-src phosphorylation and prevents the proliferation of Triple-Negative Breast Cancer (TNBC) cells which overexpress Syndecan-Binding Protein (SDCBP). PLoS ONE.

[32] Bonanno L., Costa C., Majem M., Favaretto A., Rugge M., Rosell R. (2013). The predictive value of BRCA1 and RAP80 mRNA expression in advanced non-small-cell lung cancer patients treated with platinum-based chemotherapy. Ann. Oncol..

[33] Rosell R., Perez-Roca L., Sánchez J.J., Cobo M., Moran T., Chaib I., Provencio M., Dómine M., Sala M., Jimenez U. (2009). Customized Treatment in Non-Small-Cell Lung Cancer Based on EGFR Mutations and BRCA1 mRNA Expression. PLoS ONE.

[34] Friboulet L., Olaussen K.A., Pignon J.-P., Shepherd F.A., Tsao M.-S., Graziano S., Kratzke R., Douillard J.-Y., Seymour L., Pirker R. (2013). ERCC1 Isoform Expression and DNA Repair in Non–Small-Cell Lung Cancer. N. Engl. J. Med..

[35] Mack P.C., Gandara D.R., Bowen C., Edelman M., Paglieroni T., Schnier J.B., Gelmann E.P., Gumerlock P.H. (1999). RB status as a determinant of response to UCN-01 in non-small cell lung carcinoma. Clin. Cancer Res..

[36] Kelber J.A., Reno T., Kaushal S., Metildi C., Wright T., Stoletov K., Weems J.M., Park F.D., Mose E., Wang Y. (2012). KRas Induces a Src/PEAK1/ErbB2 Kinase Amplification Loop That Drives Metastatic Growth and Therapy Resistance in Pancreatic Cancer. Cancer Res..

[37] Cooper W.A., Tran T., Vilain R.E., Madore J., Selinger C.I., Kohonen-Corish M., Yip P., Yu B., O’Toole S.A., McCaughan B.C. (2015). PD-L1 expression is a favorable prognostic factor in early stage non-small cell carcinoma. Lung Cancer.

[38] Yu H., Boyle T.A., Zhou C., Rimm D.L., Hirsch F.R. (2016). PD-L1 expression in Lung Cancer. J. Thorac. Oncol..

[39] Skoulidis F., Li B.T., Dy G.K., Price T.J., Falchook G.S., Wolf J., Italiano A., Schuler M., Borghaei H., Barlesi F. (2021). Sotorasib for lung cancers with KRAS p. G12C mutation. N. Engl. J. Med..

[40] Gandhi L., Rodríguez-Abreu D., Gadgeel S., Esteban E., Felip E., De Angelis F., Domine M., Clingan P., Hochmair M.J., Powell S.F. (2018). Pembrolizumab plus Chemotherapy in Metastatic Non–Small-Cell Lung Cancer. N. Engl. J. Med..

[41] Jänne P.A., Van Den Heuvel M.M., Barlesi F., Cobo M., Mazieres J., Crinò L., Orlov S., Blackhall F., Wolf J., Garrido P. (2017). Selumetinib plus docetaxel compared with docetaxel alone and progression-free survival in patients with kras-mutant advanced non–small cell lung cancer: The select-1 randomized clinical trial. JAMA.

[42] Garon E.B., Ciuleanu T.-E., Arrieta O., Prabhash K., Syrigos K.N., Goksel T., Park K., Gorbunova V., Kowalyszyn R.D., Pikiel J. (2014). Ramucirumab plus docetaxel versus placebo plus docetaxel for second-line treatment of stage IV non-small-cell lung cancer after disease progression on platinum-based therapy (REVEL): A multicentre, double-blind, randomised phase 3 trial. Lancet.

[43] Awad M.M., Liu S., Rybkin I.I., Arbour K.C., Dilly J., Zhu V.W., Johnson M.L., Heist R.S., Patil T., Riely G.J. (2021). Acquired resistance to KRASG12C inhibition in cancer. N. Engl. J. Med..

